# The effect of three urease inhibitors on *H. pylori* viability, urease activity and urease gene expression

**DOI:** 10.3389/fmicb.2024.1464484

**Published:** 2024-11-15

**Authors:** Hanaa Shaalan, Maya Azrad, Avi Peretz

**Affiliations:** ^1^The Azrieli Faculty of Medicine, Bar-Ilan University, Safed, Israel; ^2^Clinical Microbiology Laboratory, Tzafon Medical Center, Poriya, Israel, Affiliated with Azrieli Faculty of Medicine, Bar Ilan University, Safed, Israel

**Keywords:** *Helicobacter pylori*, urease inhibitors, urease activity, bacterial viability, urease genes

## Abstract

**Background:**

Treatment of *Helicobacter pylori* (*H. pylori*) infections is challenged by antibiotic resistance. The urease enzyme contributes to *H. pylori* colonization in the gastric acidic environment by producing a neutral microenvironment. We hypothesized that urease inhibition could affect *H. pylori* viability. This work aimed to assess the effects of acetohydroxamic acid (AHA), ebselen and baicalin on urease activity, bacterial viability and urease genes expression in *H. pylori* isolates.

**Methods:**

Forty-nine *H. pylori* clinical isolates were collected. Urease activity was assessed using the phenol red method. The urease inhibition assay assessed inhibitors' effects on urease activity. Flow cytometry assessed the effect of inhibitors on bacterial viability. Real time PCR was used to compare urease genes expression levels following urease inhibition.

**Results:**

Urease activity levels differed between isolates. Acetohydroxamic acid inhibited urease activity at a concentration of 2.5 mM. Although baicalin inhibited urease activity at lower concentrations, major effects were seen at 8 mM. Ebselen's major inhibition was demonstrated at 0.06 mM. Baicalin (8 mM) significantly reduced ATP production compared to untreated isolates. Baicalin, ebselen and acetohydroxamic acid significantly reduced *H. pylori* viability. Increased urease genes expression was detected after exposure to all urease inhibitors.

**Discussion:**

In conclusion, higher concentrations of baicalin were needed to inhibit urease activity, compared to acetohydroxamic acid and ebselen. Baicalin, ebselen and acetohydroxamic acid reduced *H. pylori* viability. Therefore, these inhibitors should be further investigated as alternative treatments for *H. pylori* infection.

## 1 Introduction

*Helicobacter pylori* (*H. pylori*) is a Gram-negative, microaerophilic, spiral-shaped bacterium, which colonizes the human gastric mucosa (Malfertheiner et al., 2023) and is present in the gut of over 50% of the world population (García et al., 2014). While the infection is often asymptomatic, chronic infection can cause gastritis, gastric ulcer, mucosa-associated lymphoid tissue (MALT) lymphoma and gastric adenocarcinoma (Diaconu et al., 2017; Kusters et al., 2006). Currently accepted treatment for *H. pylori* infections is mostly a combination of proton pump inhibitor (PPI) with two antibiotics (clarithromycin, metronidazole or levofloxacin) (Lee et al., 2022; Azrad et al., 2022). Yet, many epidemiological studies have shown increased rates of *H. pylori* antibiotic resistance in recent years, which interferes with treatment efficacy (Azrad et al., 2022; Kuo et al., 2017).

*H. pylori* produces large quantities of urease (6–10% of the total protein) which converts urea into ammonia and carbamate, enabling the bacterium to survive in the acidic gastric environment by generation of a neutral microenvironment (Ansari and Yamaoka, 2017). In addition to its role in colonization, urease mediates inflammatory responses by activating monocytes and neutrophils, which elicit damage to the gastric epithelial cells (Lee and Buck, 1996).

Urease is a heterodimer composed of the UreA and UreB subunits, with two-nickel ions bound to the active site of each dimer (Ansari and Yamaoka, 2017; Woo et al., 2021). In addition to these two structural genes, the urease gene cluster contains seven accessory genes important for enzyme activation and insertion of the nickel ions (Collins and D'Orazio, 1993).

Due to its role in pathogenesis, urease has become an important target in the search for new antimicrobial agents to treat *H. pylori* infection. Several studies have demonstrated the inhibitory effects of many compounds, including natural products and synthetic drugs, against purified *H. pylori* urease (Follmer, 2010; Hassan and Šudomová, 2017). However, little is known about their performance in clinical *H. pylori* isolates. Urease inhibitors can be a substrate structure analog, which competes with urea over the active site, or a compound that interferes with the enzymatic reaction (Upadhyay, 2012).

Taking into consideration the importance of urease enzyme and the large number of studies that aimed to investigate the inhibitory effect of different compounds on purified *H. pylori* urease, our study aimed to elucidate the knowledge in this field by measuring urease activity of different clinical *H. pylori* isolates and investigating its association with infection severity. Additionally, three known *H. pylori* urease inhibitors, including natural and synthetic compounds, which differ in their inhibition mechanism, were chosen to assess their effect on 49 *H. pylori* clinical isolates, and investigated their effect on microbial cell viability. Acetohydroxamic acid (AHA) competitively inhibits urease by forming a complex with the enzyme's nickel ions (Suenaga et al., 2023; Kafarski, 2018). It is also a drug used to treat chronic urea-splitting urinary infections. It was approved by the Food and Drug Administration (FDA) as an orphan drug for the prevention of struvite stones (Marwick, 1983). Baicalin is a component of the root and aerial part of a medical plant known as *Scutellaria baicalensis*. It is considered a non-competitive inhibitor of *H. pylori* urease that interacts with Cys321 on the mobile flap of the enzyme (Yu et al., 2015). It was also reported to reduce gastric inflammation caused by *H. pylori* infection (Shih et al., 2007). Moreover, it has anti-inflammatory, anti-allergic, anti-oxidant and neuroprotective properties (Yu et al., 2015). Ebselen is a selenoorganic compound with anti-inflammatory, antioxidant, and cytoprotective activities (Macegoniuk et al., 2023). It acts as a competitive urease inhibitor that reacts with the nickel ions and cysteine 322 in the enzyme active site (Macegoniuk et al., 2016). In addition to its anti-urease activity, its antiulcer properties were demonstrated in a rat model (Tabuchi and Kurebayashi, 1993).

## 2 Materials and methods

### 2.1 Urease inhibitors

AHA, baicalin and ebselen were purchased from Acros Organics (Geel, Belgium). AHA was dissolved in double distilled water (DDW) to create a stock solution of 20 mM. Baicalin and ebselen were dissolved in 1% dimethyl sulfoxide (DMSO) (Sigma-Aldrich, Louis, USA) to create stock solutions of 16 mM for baicalin, and 0 0.25 mM for ebselen. The prepared solutions (Table 1) were stored at −20°C until further use. The concentrations were chosen based on previous reports (Yu et al., 2015; Macegoniuk et al., 2016; Goldie et al., 1991).

**Table 1:** Tested urease inhibitor concentrations.

**Urease inhibitor**	**Concentration I (mM)**	**Concentration II (mM)**	**Concentration III (mM)**
Acetohydroxamic acid	2.5	5	10
Baicalin	2	4	8
Ebselen	0.03125	0.0625	0.125

### 2.2 Bacterial isolates

Forty-nine clinical *H. pylori* isolates were randomly chosen from the isolates bank of the clinical microbiology laboratory at the Tzafon Medical Center. The isolates had been previously isolated from gastric specimens of patients undergoing gastroscopy between January 2018 and December 2021, due to symptomatic gastroduodenal pathologies. Histological data regarding infection severity was collected from patient records. The study was approved by the Medical Center Helsinki Committee, Approval no. POR 0007-20. ATCC strain 43504 (American Type Culture Collection, USA) served as a positive control.

### 2.3 Bacterial culture

*H. pylori* isolates were grown from frozen bead stocks on modified BD Helicobacter agar (Becton Dickinson, Heidelberg, Germany), and incubated under microaerophilic conditions, at 37°C, for 7 days. Then, colonies were harvested and suspended in brain-heart infusion broth supplemented with yeast extract and 0.1% L-cysteine (BHIS) (Hy Laboratories Ltd., Rehovot, Israel) or with phosphate buffered saline (PBS) (Biological Industries, Beit-Haemek, Israel).

### 2.4 Urease activity assay

Urease activity was determined using the phenol red method (Chang et al., 2020). In brief, *H. pylori* isolates were grown on modified BD Helicobacter agar for 7 days under microaerophilic conditions at 37°C, and then diluted in PBS (Biological Industries) to achieve 1 McFarland turbidity. The assay was carried out in 96-well plates, wherein a *H. pylori* suspension (50 μl/well) was mixed with 50 μl/well urease test broth (Novamed, Jerusalem, Israel) which contained a pH indicator (phenol red) and the urease substrate (urea). In case of urease activity, the pH rises, and the solution color changes from yellow to pink. Optical density (OD) at 570 nm was measured every minute, for 20 min, using a Multiskan FC microplate reader (Thermo Scientific, Waltham, USA). Urease activity was calculated using the following equation:


$$\begin{array}{r} Urease\;activity=OD_{570\text{nm}}\;\left(max\right)-OD_{570\text{nm}}\;\left(min\right)\\ OD_{\text{max}}=OD_{\text{t}=20};OD_{\text{min}}=OD_{\text{t}=0} \end{array}$$

### 2.5 Urease inhibition assay

A bacterial suspension of one McFarland turbidity was prepared as described above. *H. pylori* isolates (50 μl) were incubated with 50 μl of each inhibitor (50 μM-20 mM) for 15 min in 96-well plates, under microaerophilic conditions, at 37°C. Urease activity was measured as described above. Control wells contained bacterial suspension and DDW (without inhibitor). Blank wells contained inhibitor and PBS.

### 2.6 Microbial cell viability assay

The BacTiter-Glo™ Microbial Cell Viability Kit (Promega, Madison, USA) was used to determine the number of viable microbial cells based on ATP quantification. *H. pylori* colonies, grown as described above, were suspended in BHIS to McFarland 1 turbidity. The bacterial suspension (50 μl) was then incubated with 50 μl of the inhibitor or 50 μl DDW (control), in triplicates, in a 96-well plate, for 24 h, under microaerophilic conditions. BacTiter-Glo™ Reagent (100 μl) was placed in each well, and luminescence was recorded using the Fluoroskan™ FL Microplate Fluorimeter and Illuminometer (Thermo Fisher Scientific, *Waltham*, USA).

### 2.7 Flow cytometry

Flow cytometry was performed to determine bacterial viability following exposure to urease inhibitors compared to untreated isolates, in representative isolates (*n* = 10). *H. pylori* suspension (100 μl) at 0.6 McFarland turbidity was incubated in a tube with 100-μl urease inhibitor for 24 h, under microaerophilic conditions. Then, tubes were centrifuged and the pellet was resuspended in 200-μl NaCl 0.9% and then diluted 10-μl suspension in 477 μl NaCl 0.9%. A mixture of 1.5 μl SYTO^®^9 green fluorescent nucleic acid stain (for live bacteria) and 1.5 μl red-fluorescent nucleic acid stain, propidium iodide (PI) (for dead bacteria) (Thermo Fisher Scientific, Waltham, Massachusetts, United States) was then mixed with the suspension, to stain the bacteria. Flow cytometry was performed using a Gallios Flow Cytometer (Beckman Coulter, Indianapolis, USA), with excitation/emission wavelengths of 488/520 nm for SYTO^®^9 and 488/>630 nm for PI.

### 2.8 Quantitative real-time PCR

Urease gene (*ureA, ureB*) expression levels in representative isolates were quantified using RT PCR. Briefly, *H. pylori* isolate suspensions in BHIS were incubated with each urease inhibitor, for 24 h, as described above. Then, total RNA was extracted from suspensions using the RNeasy Mini Kit (QIAGEN, Hilden, Germany) and then reverse-transcribed using the qPCRBIO cDNA Synthesis Kit (PCR Biosystems Inc, Pennsylvania, USA). Each real-time qPCR reaction included 10 μl PowerUp™ SYBR™ Green Master Mix (Thermo Fisher Scientific, Baltics, UAB), 0.5 μl primers (forward, and reverse, at final concentration of 500 nm each; Table 2) (Hy Laboratories), 4 ng cDNA and water to a final volume of 20 μl. The 16s rRNA gene served as a housekeeping gene. The PCR was performed using the CFX96 Real-Time PCR System (Bio Rad, California, USA), under the following conditions: for *16s* and *ureB* −95°C for 15 s, 58°C for 15 s and 72°C for 1 min, 40 cycles and for *urea* −95°C for 15 s, and 60°C for 1 min, 40 cycles. Results were analyzed using the ΔΔCt method.

**Table 2:** Primers used for real-time qPCR reactions.

**Gene**	**Forward primer (5^′^-3^′^)**	**Reverse primer (5^′^-3^′^)**
*ureA*	CGTGGCAAGCATGATCCAT	GGGT ATGCACGGTTACGAGTTT
*ureB*	TCTATCCCTACCCCACAACC	CCATCCACGAACACATGGTA
*16s*	TATGACGGGTATCCGGC	ATTCCACTTACCTCTCCCA

### 2.9 Statistical analysis

The analysis of variance (ANOVA) test was applied to identify differences in urease activity between the different isolates and between urease inhibitor-treated and untreated isolates. The ANOVA test was also applied to assess differences in ATP production and bacterial cell viability between urease inhibitor-treated vs. untreated isolates. *T*-test was applied to compare gene expression levels of urease inhibitor-treated vs. control isolates, for each inhibitor. All tests applied were two-tailed, and a *p*-value of 5% or less was consider statistically significant. The data were analyzed using the Graphpad Prism software, version 9.5.1.733 (GraphPad Software, Florida, Boston, MA).

## 3 Results

### 3.1 Urease activity of the clinical isolates and association with infection severity

**Figure 1:**
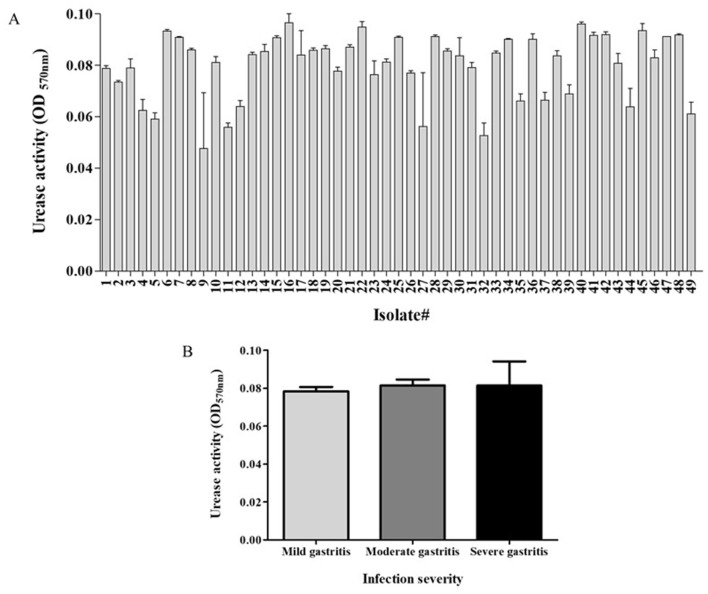
Urease activity of study's isolates and relation to disease severity. Activity of *H. pylori* urease was measured as the difference between the optical density measured in 570 nm- OD_570_ (*t* = 20 min) and initial OD_570_ (*t* = 0). **(A)** The urease activity of the different isolates. **(B)** Urease activity in relation to infection severity. Each experiment was conducted in triplicates, *n* = 49.

The clinical isolates exhibited urease activity in the range of 0.04–0.09 (arbitrary units) (Figure 1A). No significant association was found between urease activity and infection severity (Figure 1B).

### 3.2 Inhibition of urease activity in clinical isolates by the different inhibitors

#### 3.2.1 Acetohydroxamic acid

**Figure 2:**
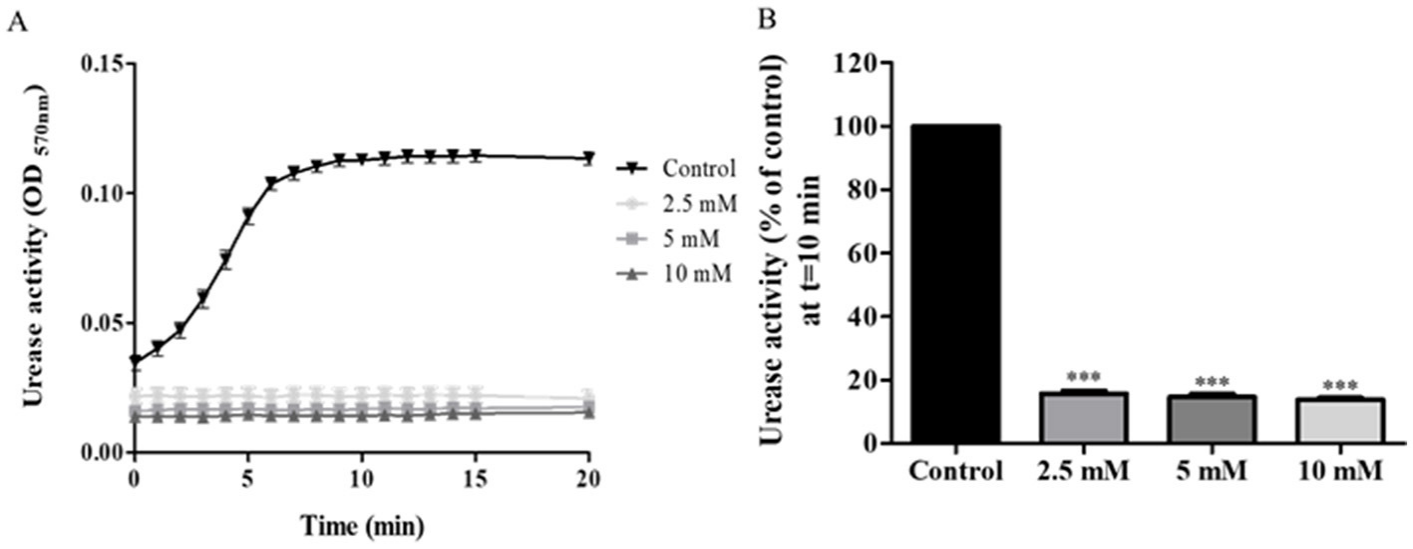
Urease activity of acetohydroxamic acid (AHA)-treated. *H. pylori* isolates were treated with different concentration of AHA for 15 min, then urease activity was assessed over 20 min by measuring OD_570nm_. **(A)** A representative graph of urease activity of one isolate, which was treated, with three different concentrations of AHA or untreated (Control). **(B)** Urease activity of 49 isolates treated for 10 min, with three different concentrations of AHA for 15 min was detected at *t* = 10 min and compared to the control (untreated). ***p < 0.001, *n* = 49. Each experiment was conducted in triplicates. AHA, acetohydroxamic acid.

Urease activity of isolates was significantly lower in AHA-treated isolates compared to untreated bacteria. Almost full inhibition (84% reduction) was already achieved at an inhibitor concentration of 2.5 mM (*p* < 0.001) (Figure 2A). There was no significant difference in the effect of the three tested concentrations on urease activity (Figure 2B).

#### 3.2.2 Baicalin

**Figure 3:**
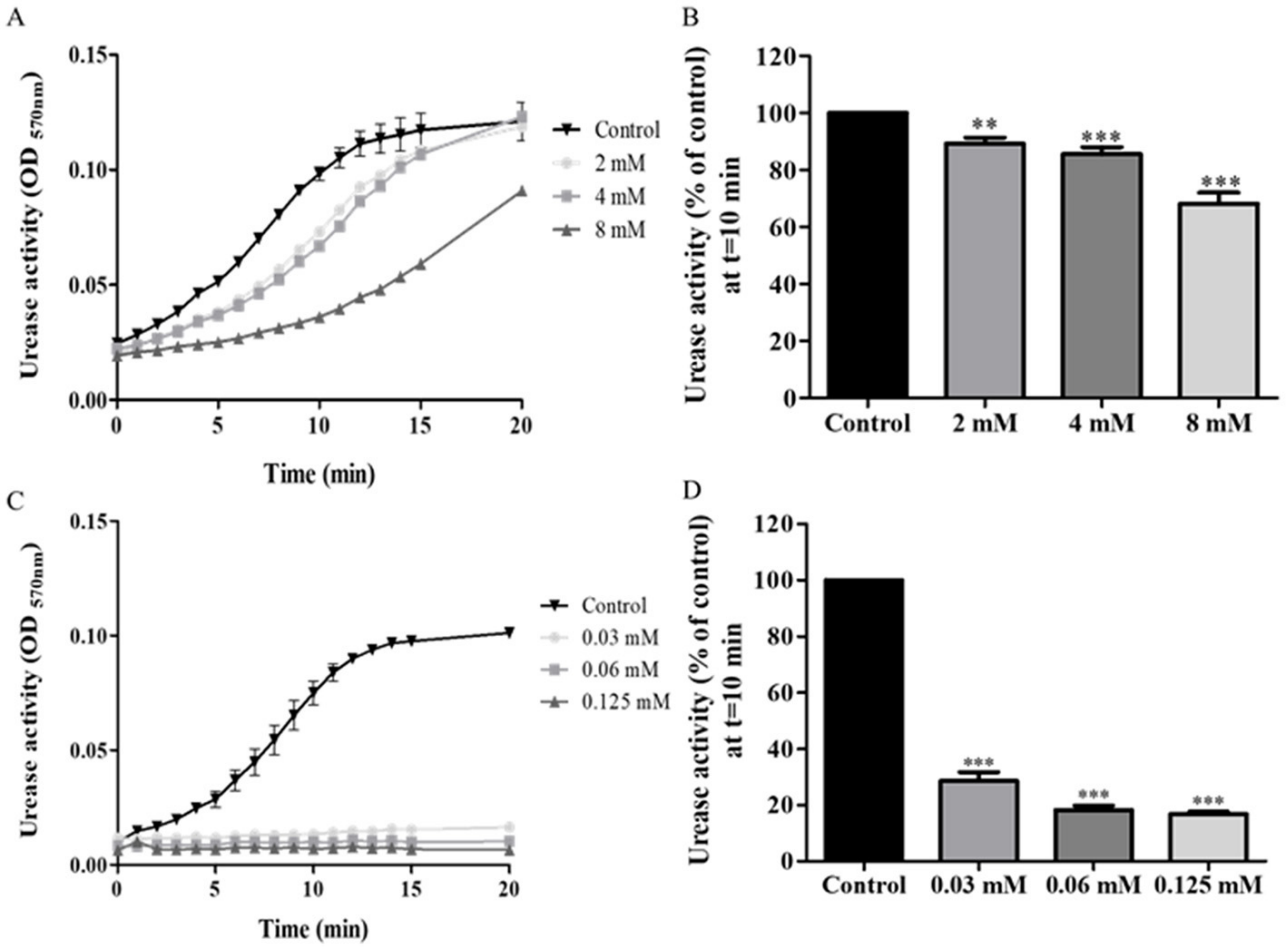
Urease activity of isolates treated with baicalin and ebselen. *H. pylori* isolates were treated with different concentration of baicalin or ebselen for 15 min, and then urease activity was detected by measuring OD at 570 nm, during 20 min. **(A)** A representative graph of urease activity of one isolate, which was treated with baicalin at three different concentrations or untreated (Control). **(B)** Urease activity of the isolates treated with three different concentrations of baicalin for 15 min was detected at *t* = 10 min and compared to the control (untreated). **(C)** A representative graph of urease activity of one isolate which was treated with ebselen at three different concentrations for 15 min or untreated (Control). **(D)** Urease activity of the isolates treated with three different concentrations of ebselen for 15 min was detected at *t* = 10 min and compared to the control (untreated), ***p < 0.001, *n* = 49. Each experiment was conducted in triplicates. **p < 0.01.

During the first 15 min of the assay, urease activity in isolates treated with 2 mM or 4 mM baicalin was lower than urease activity of controls. However, at 20 min, they reached the same levels of activity as those of controls (Figure 3A). From a concentration of 2 mM, baicalin significantly inhibited urease activity, with a 50% reduction in activity measured upon treatment with 8 mM baicalin (*p* < 0.001) (Figure 3B).

#### 3.2.3 Ebselen

Significantly reduced urease activity was measured throughout the entire assay, in all isolates treated with any of the three tested concentrations of ebselen (Figure 3C). Even at the low concentration of 0.03 mM, a 71% reduction from control was measured in urease activity (*p* < 0.001) (Figure 3D).

### 3.3 Effect of urease inhibitors on ATP production in clinical isolates

**Figure 4:**
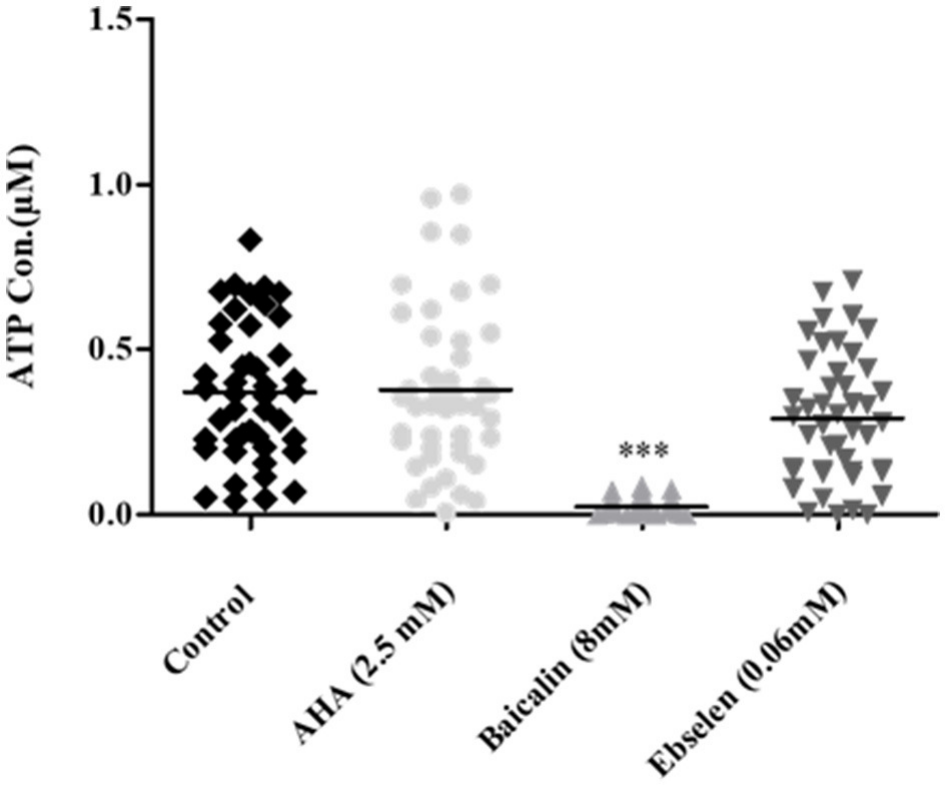
ATP production in *H. pylori* isolates treated with urease inhibitors. Isolates were treated with the minimal urease inhibitory concentration of each inhibitor (AHA = 2.5 mM, baicalin = 8 mM and ebselen = 0.06 mM) for 24 h. ATP concentration was determined by luminescence. Untreated isolates served as control. ***p < 0.001, *n* = 47. AHA, Acetohydroxamic acid.

To determine whether the urease inhibitors affect bacterial cell viability, ATP production was measured is an indicator of cell metabolism. ATP levels in isolates following incubation baicalin 8 mM were significantly lower compared to their levels in untreated control cells. In contrast, AHA 2.5 mM and ebselen 0.06 mM had no effect on bacterial cell activity (Figure 4).

### 3.4 Effect of urease inhibitors on clinical isolate cell viability

**Figure 5:**
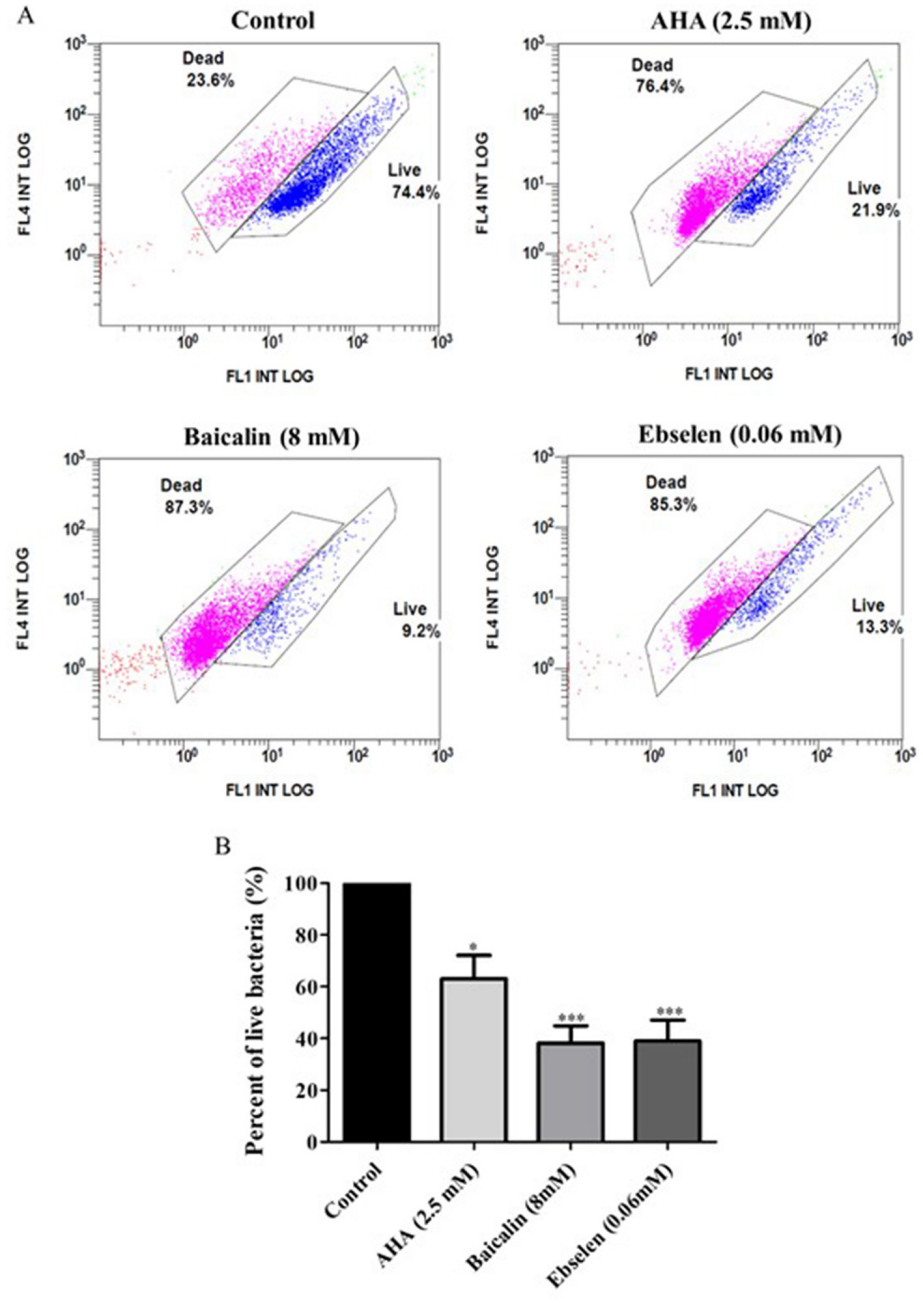
Viability of bacterial isolates treated with different urease inhibitors. Viability of bacterial isolates treated with different urease inhibitors. *H. pylori* representative isolates were incubated with urease inhibitors for 24 h (AHA = 2.5 mM, baicalin = 8 mM or ebselen = 0.06 mM) and then bacterial viability was measured by flow cytometry. The graphs in **(A)** present viable (blue) and dead (pink) cells of Control (untreated isolates), 2.5 mM AHA-treated isolates, 8 mM baicalin-treated isolates, and 0.06 mM-ebselen treated isolates. Propidium iodide, which stains dead bacteria, was detected by the FL4 channel, and SYTO9, which stains live bacteria, was detected by the FL1 channel. The graph in **(B)** present the percent of live bacteria in isolates treated with urease inhibitors, compared to control. *p < 0.05, ***p < 0.001, *n* = 10. AHA, acetohydroxamic acid.

Exposure to urease inhibitors significantly decreased the percentage of live bacteria compared to untreated bacteria (Figure 5A; Table 3). For example, exposure to AHA resulted in 21.9% live cells, as compared to the control untreated strains, in which 74.4% of the cells were viable. The greatest decrease (61.82% decrease and 60.97% decrease, respectively) in the percentage of viable bacteria was observed following baicalin and ebselen treatment (*p* < 0.001) (Figure 5B).

**Table 3:** Effect of urease inhibitors on bacterial cell viability.

**Control**	**AHA (2.5 mM)**	**Baicalin (8 mM)**	**Ebselen (0.06 mM)**
100%	63.5%	26.68%	38.6%
100%	67%	47.62%	25.21%
100%	63.36%	28%	79.13%
100%	7.811%	5.6%	19.76%
100%	74.42%	38.24%	67.33%
100%	87.47%	54.92%	74.29%
100%	15.02%	31.68%	8.64%
100%	87.03%	44.76%	35.02%
100%	80.03%	21.05%	27.78%
100%	84.52%	83.25%	14.55%

### 3.5 Urease gene expression following exposure of clinical isolates to urease inhibitors

**Figure 6:**
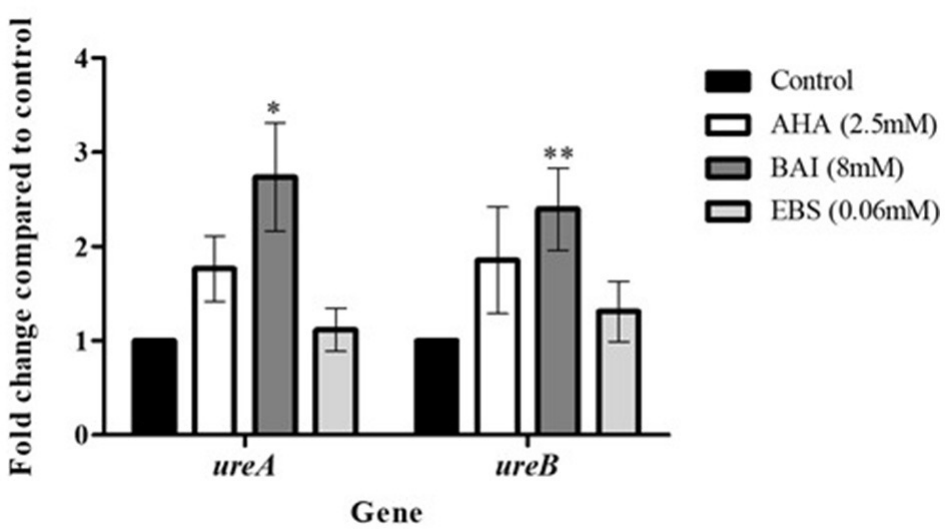
Urease genes expression following exposure to urease inhibitors. Urease genes expression following exposure to urease inhibitors. *H. pylori* isolates were treated with urease inhibitors for 24 h, then RNA was extracted and real-time PCR was performed to detect the expression levels of *ureA, ureB* and the housekeeping gene *16s*. *p < 0.05, ** *p* < 0.01, *n* = 13. AHA, acetohydroxamic acid; BAI, Baiclain; EBS, Ebselen.

*ureA* and *ureB* gene expression was upregulated in cells exposed to urease inhibitors. However, this increase was only statistically significant in baicalin-treated isolates, where 3-fold increase compared to control were measured for both genes (*p*<*0.05*) (Figure 6).

## 4 Discussion

Antibiotic resistance poses an increasing challenge to the effectiveness of *H. pylori* treatment. The search for alternative treatments has become crucial and many researchers have focused on urease inhibition (Woo et al., 2021; Kafarski, 2018; Svane et al., 2020). In contrast to previous studies that investigated urease inhibitors on enzymes, mostly purified from a limited number of strains, the present study assessed the effects of three inhibitors on urease enzymes of 49 clinical isolates.

### 4.1 Urease activity and infection severity

While the different isolates exhibited different levels of urease activity, no association was found between urease activity and infection severity. In contrast, previous studies showed a correlation between urease activity and disease severity (Ito et al., 1995; Ghalehnoei et al., 2016; Igarashi et al., 2001). For example, one study found higher urease activity in *H. pylori* strains isolated from patients with intestinal metaplasia, compared to isolates from patients with peptic ulcer disease (Ghalehnoei et al., 2016). Another study measured higher urease activity in *H. pylori* isolates from oncology patients compared to than isolates from duodenal ulcer patients (Ito et al., 1995). Although no such association was found here, a correlation may exist between the disease severity and other virulence factors, such as Cag A and Vac A as presented in other studies (Ghalehnoei et al., 2016; Roshrosh et al., 2023).

### 4.2 Inhibition of urease activity by AHA, ASA, baicalin and ebselen

This study focused on three urease inhibitors. AHA is a competitive inhibitor that forms a complex with nickel ions in the metallo-center of the enzyme (Suenaga et al., 2023; Kafarski, 2018) and is used to treat chronic urea-splitting urinary infections. Zhou et al. (2017) ound that a concentration of 0.07 mM AHA was needed to achieve 50% urease inhibition. Another study reported that the ideal AHA inhibition concentration was 2.6 mM (Goldie et al., 1991), almost the same as the concentration found most effective in the current study.

Baicalin has anti-inflammatory, anti-allergic and anti-oxidant properties. The compound is a non-competitive inhibitor which interacts with Cys321 on the mobile flap of urease (Yu et al., 2015). A previous work measured 0.82 mM as the IC_50_ of baicalin toward purified *H. pylori* urease, a concentration much lower than the present results (Yu et al., 2015).

Ebselen is a competitive inhibitor, reacting with the nickel ions and cysteine322 (Macegoniuk et al., 2016), and induces anti-inflammatory, antioxidant and cytoprotective effects. Biernat et al. found reported that the IC_50_ of ebselen toward *H. pylori* urease at time zero was 45 and 3.69 μM if the cells were preincubated with ebselen for 2 h (Macegoniuk et al., 2016). The authors concluded that the difference in the concentrations needed for inhibition was due to the time needed for diffusion of the inhibitor through the bacterial membrane. This finding underscores the importance of the present analysis of clinical isolates, as most previous studies tested the effects of inhibitors on purified urease.

The inhibition mode of each compound was reflected in the results. AHA and ebselen, both competitive inhibitors, achieved almost full inhibition of urease, even at very low concentrations, with no significant differences measured between the different tested inhibitor concentrations. In comparison, the urease inhibition capacity of baicalin, a non-competitive inhibitor, differed at the three tested concentrations, with significant urease inhibition achieved only at high concentrations. Interestingly, the effect of the inhibitors was not uniform across all the 49 isolates; in some cases, the treatment had a noticeable inhibitory effect on some isolates but no effect on other isolates. For example, after treatment with 2.5 mM AHA, < 80% urease inhibition was measured in 10 isolates, 80–90% inhibition in 29 isolates and above 90% inhibition in 10 isolates. These differences may be associated with the expression and activities of other virulence factors of the bacterium, which protect against urease inhibition.

### 4.3 Effect of urease inhibitors on bacterial cell viability

Several studies assessed the effect of urease inhibitors on *H. pylori* growth (Woo et al., 2021), morphology (Tran Trung et al., 2020), viability (Goldie et al., 1991), and adhesion to gastric epithelial cells (Chang et al., 2020). In the present study, the lowest inhibitor concentration that showed a significant inhibitory effect on enzyme activity, was applied to test its effect on *H. pylori* viability. Ebselen and AHA, which had a notable inhibitory effect at very low concentrations, did not reduce ATP production, but did reduced bacterial cells viability. In contrast, baicalin, which only inhibited urease activity at high concentrations, significantly reduced ATP production. From these results, it can be concluded that ATP production is not necessarily an indicator of cell viability and that urease inhibition does not necessarily correlate with *H. pylori* eradication. Exposure to stressful conditions may have different effects on bacterial community; on one hand, it can reduce bacterial viability, however on the one hand it may alter a survival mechanism to produce a large amount of ATP. Thus, in order to choose an effective urease inhibitor for *H. pylori* infection treatment, its effect on bacterial cell viability should be taken into consideration.

### 4.4 Effect of urease inhibition on urease gene expression levels

*H. pylori* urease consists of six copies of two structural subunits (*Ure*A and *Ure*B), with two nickel ions in the UreB subunit, in addition to six accessory subunits (*Ure*E, *Ure*F, *Ure*G, *Ure*I, *Ure*D) (Mobley et al., 1995). Examination of the effect of urease inhibition on *ureA* and *ureB* gene expression found that only baicalin significantly increased gene expression. Such upregulation may be a bacterial mechanism to compensate for inhibited urease activity.

## 5 Conclusions

In this study, we showed that the three compounds, AHA, baicalin and ebselen have an inhibitory effect on *H. pylori* urease. AHA and ebselen were more potent compared to baicalin. Baicalin, AHA, and ebselen significantly reduced *H. pylori* viability, and further study should investigate their usefulness for eradication of *H. pylori* infection. Additional studies are still needed to assess the effect of these inhibitors on human host cells.

## 6 Limitations of the study

One limitation of this study is the narrow spectrum of inhibitors concentrations; it is important to check more concentrations to assess the maximum and minimum inhibitory concentration of each inhibitor. Moreover, no additional pathogenic factors were taken into account in this study, for example, we didn't check correlations of our results with CagA and flagella. Future studies are needed to check these correlations.

## Data Availability

The original contributions presented in the study are included in the article/supplementary material, further inquiries can be directed to the corresponding author.
